# The emerging role of long non-coding RNAs in schizophrenia

**DOI:** 10.3389/fpsyt.2022.995956

**Published:** 2022-09-26

**Authors:** Guangxian Wu, Xinzhe Du, Zexuan Li, Yanhong Du, Jinzhi Lv, Xinrong Li, Yong Xu, Sha Liu

**Affiliations:** ^1^Shanxi Key Laboratory of Artificial Intelligence Assisted Diagnosis and Treatment for Mental Disorder, First Hospital of Shanxi Medical University, Taiyuan, China; ^2^Department of Physiology, School of Basic Medical Sciences, Shanxi Medical University, Taiyuan, China; ^3^Department of Psychiatry, First Hospital/First Clinical Medical College of Shanxi Medical University, Taiyuan, China; ^4^Department of Mental Health, Shanxi Medical University, Taiyuan, China

**Keywords:** schizophrenia, long non-coding RNAs, pathophysiology, biomarker, therapy

## Abstract

Schizophrenia (SZ) is a severe psychiatric disorder which is contributed by both genetic and environmental factors. However, at present, its specific pathogenesis is still not very clear, and there is a lack of objective and reliable biomarkers. Accumulating evidence indicates that long non-coding RNAs (lncRNAs) are involved in the pathophysiology of several psychiatric disorders, including SZ, and hold promise as potential biomarkers and therapeutic targets for psychiatric disorders. In this review, we summarize and discuss the role of lncRNAs in the pathogenesis of SZ and their potential value as biomarkers and therapeutic targets.

## Introduction

Schizophrenia (SZ) is a severe psychiatric disorder characterized by the incoordination of mental activities, which involves disorders of sensory perception, thinking, emotion, behavior, and so on (1). The disease typically presents in late adolescence or early adulthood with a lifetime prevalence of approximately 1% (2), a protracted disease course with high recurrence and disability rates, and a substantial disease burden for families and society (1). Because SZ is a complex and heterogeneous disorder, its exact pathogenesis has been unclear so far. It is generally accepted that the occurrence and development of SZ result from a combination of genetic and environmental factors, and multiple pathophysiological mechanisms may be involved (1). These include neurotransmitter system abnormalities (3), neurodevelopmental disorders (4), immune dysfunction (5), redox imbalance (6), glial cell dysfunction (7), and gut microbiota alterations (8). In addition, the clinical diagnosis of SZ mainly relies on symptomatology, lacks objective and reliable biomarkers, and is highly prone to underdiagnosis and misdiagnosis (1).

Long non-coding RNAs (lncRNAs), a class of RNA transcripts greater than 200 nucleotides in length without protein coding capacity, were once considered as transcriptional noise (9). Most lncRNAs, like mRNAs, are transcribed by RNA polymerase II (Pol II) and are capped, polyadenylated and alternatively spliced. However, lncRNAs function is closely related to its subcellular localization (10, 11). For example: In the nucleus, lncRNAs regulate the structure and function of chromatin by interacting with chromatin and regulating the transcription and alternative splicing of genes by interacting with transcription factors and splicing factors. In the cytoplasm, lncRNAs play a role of post transcriptional regulation by regulating the stability and translation of mRNAs and post-translational modifications. In mitochondria, lncRNAs not only regulate mitochondrial structure and function, but also play an important role in the reciprocal communication between mitochondria and the nucleus. In exosomes, lncRNAs play a role in information communication between cells (12).

Recent studies have reported that lncRNAs have remarkable spatiotemporal expression profiles and exhibit highly specific expression patterns in the brain (13), which have been reported to function as regulatory molecules involved in the pathogenesis of central nervous system (CNS) disorders including psychiatric disorders (14). For example: in depression, lncRNAs can mediate pathogenesis by regulating various pathways such as neurotransmitters and neurotrophic factors and affecting synaptic transmission (15), in autism spectrum disorder, by regulating various pathways such as immune responses, neural development and synaptic transmission (16), and in bipolar disorder, by regulating numerous pathways such as neuroinflammation, prominent plasticity and cell proliferation (17). Meanwhile lncRNAs can be detected in various types of body fluids of human body with good availability, which are considered as potential biomarkers and therapeutic targets for CNS disorders (18). In this review, we summarize and discuss the role of lncRNAs in the pathogenesis of SZ and their potential value as biomarkers and therapeutic targets.

## Role of long non-coding RNAs in the pathogenesis of schizophrenia

Recently, several studies have identified differentially expressed lncRNAs in the peripheral circulating blood and postmortem brain tissues of patients with SZ, and some of them performed bioinformatics analysis of these differentially expressed lncRNAs. Ren et al. found differentially expressed lncRNAs associated with mitochondrial function in peripheral blood of 19 drug-naive early-onset SZ (EOS) patients and 18 healthy control subjects (19). Hu et al. found no differentially expressed lncRNAs in postmortem orbitofrontal cortex (BA11) of 16 SZ patients and 12 healthy control subjects, 34 and 1 differentially expressed lncRNAs each in postmortem anterior cingulate cortex (BA24) and postmortem dorsolateral prefrontal cortex (BA9) of 6 SZ patients and 6 healthy control subjects. Bioinformatics analysis identified these differentially expressed lncRNAs involved in immune system, brain development, myelination, and oligodendrocyte differentiation (20). In addition, there are two other studies exploring the expression of lncRNAs and their potential roles in the amygdala of patients with SZ. Liu et al. found 250 differentially expressed lncRNAs in the amygdala region of 22 SZ patients and 24 healthy control subjects, among which RP11-677M14.2 showed decreased expression in the paranoid subtype and RP11-724N1.1 showed increased expression in the undifferentiated subtype (21). Tian et al. identified 110 differentially expressed lncRNAs in the amygdala region of 21 SZ patients and 24 healthy control subjects, and subsequently classified 21 SZ patients into five paranoid, seven disorganized, and nine undifferentiated. Compared with controls, 171 differentially expressed lncRNAs were found in the undifferentiated subtype, 45 differentially expressed lncRNAs were found in disorganized subtype, and no differentially expressed lncRNAs were found in the paranoid subtype. Bioinformatics analysis identified that these differentially expressed lncRNAs were related to synaptic transmission, ribosome function and immune function (22). Taken together, it is suggested that lncRNAs may not only be involved in the pathogenesis of SZ, but also can be used to distinguish subtypes of SZ. In addition, the results of the bioinformatics analysis deserve further experimental verification in the future.

In addition to the results of bioinformatics analysis, related basic experimental results further indicated that lncRNAs play an important role in the pathogenesis of SZ, including the involvement of immune function, alternative splicing and expression of genes, etc. (Table 1). As the roles of lncRNAs in the pathogenesis of SZ continue to be decoded, this will provide a valuable theoretical basis for subsequent lncRNAs as potential biomarkers and therapeutic targets for this disease.

**TABLE 1:** Studies that investigated the role of lncRNAs in schizophrenia.

LncRNAs	Samples	Changes/ Polymorphism	Source	Function	References
IFNG-AS1	27 SZ and 32 healthy controls	Down	PBMCs	The expression level of IFNG-AS1 was positively correlated with that of IFNG.	(25)
AC006129.1	Monozygotic twins discordant for SZ	Up	Peripheral blood	AC006129.1 Binds to the promoter region of the transcriptional repressor CIC, facilitates the interactions of DNA methyltransferases with the CIC promoter, and promotes DNA methylation-mediated CIC downregulation, thereby ameliorating CIC-induced SOCS3 and CASP1 repression.	(27)
RP5-998N21.4	Monozygotic twins discordant for SZ	Up	Peripheral blood	RP5-998N21.4 Promotes immune defense by upregulating IFIT2 and IFIT3.	(28)
Gomafu	28 SZ and 28 healthy controls	Down	Superior temporal gyrus	Gomafu can bind to multiple splicing factors and participate in the alternative splicing of DISC1 and ERBB4.Gomafu knockout mice exhibit mild hyperactivity and anxiety-like behavior.	(32, 39, 40)
DGCR5	259 BrainGVEX control brain samples and 37 Developmental Capstone samples from the developing human brain	CNV	Brain	DGCR5 regulates the expression of certain SZ-related genes.	(34)
EU358092	GWAS, GenBank, ENCODE	within the schizophrenia-associated region at 1p21.3	Brain	Expression of EU358092 is regulated by psychotropic drugs treatment in the human SH-SY5Y neuroblastoma cell line, and EU358092 and MIR137 are commonly co-expressed *in vivo*.	(35)
NEAT1	28 SZ and 22 healthy controls	Down	Multiple cortical areas	NEAT1 is involved in the regulation of oligodendrocyte function.	(37)
NONHSAT089447	106 SZ and 48 healthy controls	Up	PBMCs	NONHSAT08944 regulates dopamine receptor expression.	(42)
Gomafu	1,093 SZ and 1,180 healthy controls	rs1894720, rs4274	Peripheral blood leukocytes	There was a significant association of rs1894720 with paranoid SZ in the Chinese Han population, and a weak association of rs4274 with paranoid SZ in the Chinese Han population.	(43)
LINC00461	GWAS, 32 first-onset SZ and 48 healthy controls	rs410216	peripheral blood cells	rs410216 was significantly associated with risk of SZ, and subjects carrying the rs410216 risk allele exhibited reduced volume and lower linc00461 transcript levels in the hippocampus.	(44)
linc01080	1139 SZ and 1039 healthy controls	rs7990916	Peripheral blood	rs7990916 may affect the age of onset and neurocognitive function of patients with SZ.	(46)
LOC105372125	548 SZ and 598 healthy controls	rs12966547	Peripheral blood	rs12966547 was significantly associated with the risk of SZ in Han Chinese women, but not in Han Chinese men.	(47)

### Long non-coding RNAs involved in immune function

Immune dysregulation plays an important role in the pathogenesis of SZ (23). A meta-analysis identified dysregulated cytokine expression in peripheral blood in SZ, which includes interferon-gamma (IFNG) (24). Ghafelehbashi et al. found that lncRNA IFNG-AS1 expression was decreased in peripheral blood mononuclear cells (PBMCs) of SZ patients and positively correlated with IFNG expression level (25). In fact, previous studies have found that IFNG-AS1 can promote IFNG expression through the mechanism of histone methylation (26).

Ni et al. found upregulated lncRNA-AC006129.1 expression in the peripheral blood of monozygotic twins discordant for SZ (27). AC006129.1 overexpressing mice exhibited abnormal behaviors associated with SZ, AC006129.1 was further found to bind to the promoter region of the transcriptional repressor Capicua (CIC), facilitating DNA methyltransferases to interact with the CIC promoter region and resulting in downregulation of CIC expression, thereby reversing CIC mediated inhibition of expression of SOCS3 and CASP1. Increased expression of SOCS3 may enhance anti-inflammatory responses by inhibiting JAK/STAT signaling activation, whereas increased expression of CASP1 may enhance pro-inflammatory responses by promoting interleukin-1β (IL-1β) secretion (27). This result indicated that AC006129.1 regulated not only pro-inflammatory response but also anti-inflammatory response, and this regulation pattern would provide a new idea for the study of the pathogenesis of SZ. Notably, the team identified an additional lncRNA RP5-998N21.4 with upregulated expression in the peripheral blood samples of monozygotic twins discordant for SZ, and further experiments found that RP5-998N21.4 is required for enhancing interferon-induced protein with tetratricopeptide repeat (IFIT) 2- and IFIT3-mediated immune defense responses by activating the signal transducer and activator of transcription 1 (STAT1) signaling pathway (28).

### Long non-coding RNAs involved in regulating alternative splicing and expression of genes

Aberrant alternative splicing has been reported in SZ in several genes related to neurodevelopment and neurological function (29), including DISC1 (disrupted in schizophrenia 1) and ErbB4 (Erb-B2 receptor tyrosine kinase 4) (30, 31). Recent studies have found that the gene for lncRNA Gomafu (also known as MIAT and RNCR2), which is located in the chromosome 22q12.1 region implicated in SZ, is downregulated in cortical gray matter of the postmortem superior temporal gyrus of patients with SZ (32), but elevated in PBMCs (33). Barry et al. found that Gomafu can bind to multiple splicing factors, and dysregulated expression of Gomafu would lead to DISC1 gene and ErbB4 gene occurrence similar to the aberrant alternative splicing pattern found in SZ (32).

Meng et al. identified lncRNA DGCR5 in the chromosome 22q11.2 copy number variation (CNV) regions associated with the risk of SZ. Subsequent knockdown and overexpression of DGCR5 in human neural progenitor cells derived from human induced pluripotent stem cells, DGCR5 was found to regulate the expression of several SCZ-related genes (34). In addition, Gianfrancesco et al. identified a novel lncRNA EU358092 in chromosome 1p21.3 that was highly associated with SZ. EU358092 expression was found to be regulated by psychosine in human SH-SY5Y neuroblastoma cell line, and co-expressed with MIR137 involved in SZ pathogenesis *in vivo*. MIR137 is located in the same chromosomal region as EU358092, suggesting that EU358092 may regulate MIR137 expression (35).

### Other long non-coding RNAs-based molecular links in schizophrenia

LncRNAs can also contribute to the pathogenesis of SZ through other mechanisms of action. The first mechanism of action is to mediate the oligodendrocyte dysfunction present in SZ and the myelin defects they produce (36): lncRNA NEAT1 expression was downregulated in multiple cortical regions of SZ and in peripheral blood (37, 38). Knockout of NEAT1 in mice affected the expression of multiple genes involved in oligodendrocyte differentiation and was accompanied by a decrease in the number of OLG-lineage cells in the frontal cortex, suggesting that NEAT1 is involved in OLG functions, including myelination (37). A second mechanism of action may mediate anxiety-like behaviors and mildly hyperactive behaviors associated with SZ: Knockdown of Gomafu in the medial prefrontal cortex of mice increases anxiety-like behaviors. Further experiments revealed that Gomafu represses beta crystallin (Crybb1) expression by interacting with the polycomb repressive complex 1 (PRC1) in the Crybb1 promoter region to reduce anxiety-like behaviors in mice (39). In addition, Gomafu knockout mice also showed mild hyperactivity behaviors. This hyperactive behavior was enhanced when knockout mice were treated with the psychostimulant methamphetamine, which was associated with an increase in dopamine release in the nucleus accumbens. In addition, RNA sequencing analysis identified several genes whose expression is regulated by Gomafu, and a subset of these genes have important neurobiological functions (40). A third mechanism of action is to regulate the dopaminergic system, which is closely related to SZ: The expression level of lncRNA NON-HSAT089444 was significantly upregulated in the PBMCs of SZ patients (41), and knockdown of NON-HSAT08944 was found to decrease the expression of dopamine receptors (DR) D3 and DRD5 in cytology experiments, while overexpression of NON-HSAT08944 upregulated DRD3 and DRD5 expression, suggesting that NON-HSAT08944 is involved in dopamine signaling pathway *via* upregulation of DRDs (42).

### Long non-coding RNAs single-nucleotide polymorphisms and schizophrenia

In recent years, several studies have found that single-nucleotide polymorphisms (SNPs) within lncRNAs-coding genes involved in the pathogenesis of SZ are associated with the risk of SZ. Rao et al. conducted a two-stage (discovery sample and replication sample) association analysis of eight tag SNPs covering the entire lncRNA MIAT locus in Han Chinese SZ case-control cohorts and found a significant association of rs1894720 of MIAT with paranoid SZ in a Chinese Han population, whereas rs4274 had a weak association (43). In addition, they showed that LINC00461 expression levels in peripheral blood cells of first-episode SZ patients were significantly lower than those in healthy controls before and after 12 weeks of treatment, rs410216 of LINC00461 was significantly associated with the risk of SZ. And subjects carrying the risk allele of rs410216 exhibited reduced volume and lower LINC00461 transcript levels in the hippocampus. It is suggested that LINC00461 underexpression in the hippocampus is associated with the development of SZ and can be a therapeutic target (44). In addition, LINC00461 has been identified as a pleiotropic gene for major mental disorders (45).

Qi et al. found in a Chinese Han population that rs7990916 of linc01080 was significantly associated with the age of onset of SZ patients, and patients with the T allele had an earlier age of onset than CC genotype carriers. In terms of cognitive function, patients with the T allele had lower cognitive scores than CC genotype carriers. It is suggested that rs7990916 of linc01080 may affect the age of onset and cognitive function of patients with SZ (46). Zhao et al. found that rs12966547 of LOC105372125 was significantly associated with the risk of SZ and bipolar disorder (BD) in Han Chinese women, but not in Han Chinese men. Suggested that rs12966547 of LOC105372125 is a common genetic variant for SZ and BD in Han Chinese women (47).

## Long non-coding RNAs as biomarkers in schizophrenia

In recent years, to explore whether lncRNAs have potential as biomarkers for SZ, several studies have focused on exploring the expression of lncRNAs in the peripheral circulating blood of patients with SZ (Table 2). Chen et al. found 125 differentially expressed lncRNAs, of which 62 were upregulated and 63 were downregulated, from 106 SZ patients who had not taken any antipsychotic drugs for at least 3 months and 48 matched healthy controls. It is worth noting that the expression levels of NON-HSAT089447, NON-HSAT021545 and NON-HSAT041499 were significantly upregulated in SZ patients. The expression levels of NON-HSAT089447 and NON-HSAT041499 were significantly decreased after 6 weeks of antipsychotic treatment, And the downregulation of NON-HSAT041499 expression was significantly associated with the improvement of positive and active symptoms after treatment (41). It was further found that these three up-regulated lncRNAs could serve as potential biomarkers to distinguish SZ from major depressive disorder (MDD) and generalized anxiety disorder (GAD) (48).

**TABLE 2:** LncRNAs expression as biomarkers in schizophrenia.

LncRNAs	Samples	Source	Expression	Clinical significance	References
NONHSAT089447, NONHSAT021545, NONHSAT041499	106 SZ and 48 healthy controls	PBMCs	Up	Down-regulation of NONHSAT089447 and NONHSAT041499 expression after treatment, down-regulation of NONHSAT041499 expression is correlated with symptom improvement in SZ patients.	(41)
PNKY	50 SZ and 50 healthy controls	Peripheral blood	Up	AUC = 0.78.	(49)
CHAST, CEBPA, DICER1-AS1, H19, HNF1A-AS1	50 SZ and 50 healthy controls	Peripheral blood	Up	CHAST: AUC = 0.79, CEBPA: AUC = 0.948, DICER1-AS1: AUC = 0.676, H19: AUC = 0.815, HNF1A-AS1: AUC = 0.79	(52)
SNHG6, LINC00346, LINC00511	50 SZ and 50 healthy controls	Peripheral blood	Up	SNHG6: AUC = 0.932, LINC00346: AUC = 0.795, LINC00511: AUC = 0.706.	(50)
HOXA-AS2, Linc-ROR, MEG3, SPRY4-IT1, UCA1	60 SZ and 60 healthy controls	Peripheral blood	Up only in female patients	Combination of Linc-ROR, MEG3, SPRY4-IT1 and UCA1 expression levels could differentiate female patients with 95.2% sensitivity, 76.9% specificity and diagnostic power of 0.88.	(54)
FAS-AS1, PVT1, UG1, GAS5, NEAT1, OIP5-AS1, THRIL	50 SZ and 50 healthy controls	Whole venous blood	Down (FAS-AS1, PVT1, TUG1), Up (THRIL)	Diagnosis of SZ in male persons: FAS-AS1: AUC = 0.825,TUG1: AUC = 0.832,PVT1: AUC = 0.83. Diagnosis of SZ in female persons: GAS5: AUC = 0.93, NEAT1: AUC = 0.86,OIP5-AS1: AUC = 0.87, THRIL: AUC = 0.817.	(53)
Gomafu, AK096174, AK123097, DB340248, uc011dma.1, ENST00000509804-1, ENST00000509804-2	48 SZ and 49 healthy controls	PBMCs	Down (AK096174, AK123097, DB340248, ENST00000509804-1, ENST00000509804-2) Up (Gomafu, uc011dma.1)	The combined diagnostic value of AK096174 and ENST0000509804-1: AUC = 0.925. The expression levels of AK123097, uc011dma.1 and ENST00000509804-1 were reversed after treatment, which correlated with SZ severity.	(33)
Neat1, Neat2	9 untreated SZ?9 SZ and 9 healthy controls	Peripheral blood	Down	The expression levels of Neat1 and Neat2 were increased after treatment.	(38)
MEG3, PINT, GAS5	86 Psychosis (63 SZ and 23 BD) and 44 healthy controls	PBMCs	Down (PINT, GAS5), Up (MEG3)	The expression level of MEG3 decreased after treatment, while the expression level of PINT and GAS5 did not change.	(51)

Others have explored the diagnostic and prognostic value of nine and four lncRNAs in the plasma and peripheral blood of patients with SZ, respectively. It was found that some of these lncRNAs could serve as biomarkers for the diagnosis and prognosis of SZ (33, 38). In addition, some studies have found that lncRNAs related to brain-derived neurotrophic factor (BDNF), nuclear factor-κB (NF-κB) signaling pathway, Vitamin D receptor (VDR), and heterochromatin function in immune cells can be used as diagnostic biomarkers in the peripheral blood of SZ patients (49–52). At the same time, there are some lncRNAs based on sex-specific dysregulation in the peripheral blood of SZ patients, and these lncRNAs can be used as diagnostic biomarkers (53, 54).

## The prospect of long non-coding RNAs as therapeutic targets in schizophrenia

LncRNAs with key roles in the pathogenesis of SZ may hold promise as potential therapeutic targets. In addition, lncRNAs are lower than proteins in eliciting an immune response in the body, and low expression levels, lack of translation and rapid turnover may contribute to faster therapeutic effects at lower doses (55). However, no studies have reported the therapeutic effects of targeting lncRNAs in SZ. This section briefly describes the preclinical research and clinical research progress of the currently popular lncRNAs targeting methods (Small interfering RNAs (siRNAs), antisense oligonucleotides (ASOs) and CRISPR/Cas9), and provides a reference for subsequent lncRNA-based drugs development.

SiRNAs are double stranded RNA (dsRNA) molecules consisting of 20–25 base pairs that can complement target lncRNAs and then recruit the arginine-containing RNA-induced silencing complex (RISC) to promote lncRNAs degradation. In the study of lncRNAs, siRNAs have been applied in multiple preclinical models to explore the therapeutic implications of targeting lncRNAs in various diseases (56–58). However, siRNAs still face a major challenge: off-target. Although chemical modification and low doses of siRNA can alleviate this problem, off-target effects remain (59). With the approval of five siRNAs drugs that appear sequentially worldwide (60–64), siRNA drugs targeting lncRNAs may enter a whole new era.

ASOs are a single-stranded DNA oligos, and ASOs are able to interfere with the level of target sequences in many ways, among which one of the most important interference pathways of ASOs is binding to target RNAs, forming a DNA-RNA complex and then recruiting RNase H to degrade RNAs (65). In recent years, preclinical studies of ASOs targeting lncRNAs are thriving (66–68). In addition, the FDA approved the clinical trial of Andes-1537, an ASO to achieve solid tumor treatment by targeting antisense non-coding mitochondrial RNA (ASncmtRNA) in mitochondrial long non-coding RNA (mtlncRNA), and phase I clinical results showed that Andes-1537 was well tolerated, but it was discontinued (the second part was terminated to redefine the new protocol) (69). Recently, relevant studies have conducted more in-depth studies on the mechanism of action of ASncmtRNA, laying a foundation for further targeting ASncmtRNA for rational clinical application (70). in addition, several ASO drugs for the treatment of neurodegenerative diseases are currently approved for marketing, but all are administered by invasive intrathecal methods (71). But recently developed a cholesterol modified ASO that can cross the blood-brain barrier (BBB) and inhibit the expression of target genes in brain and spinal cord cells by subcutaneous or intravenous injection (72), the findings overcome the difficulty of targeting ASOs to the CNS and bring new hope for the future treatment of CNS diseases by ASOs. Indeed the study detected ASO drugs in the liver and kidney (72), which is also a technical issue with current ASOs: off-target. Further technological advances are still needed in the future.

The CRISPR/Cas9 system is a heritable adaptive immune defense system in prokaryotes. It contains two important components, a single-stranded guide RNA (sgRNA) and the Cas9 endonuclease. The sgRNA complementary to the target sequence guides the Cas9 endonuclease to cleave the specifically targeted DNA (73). Two DNA repair mechanisms are then utilized to repair the broken DNA, namely non-homologous end-joining (NHEJ) or homology-directed repair (HDR). With these two repair mechanisms, insertion and knockout of genes can be achieved (74). Currently, the therapeutic effects of CRISPR/Cas9 targeting lncRNAs are still limited to basic research (75, 76). Among them, off-target effects and lack of safe and efficient targeted delivery vehicles are the key factors that constrain their clinical translation (77). In response to these issues, recently, studies have dissected the structural features of CRISPR/Cas9 to generate off-target effects, based on which Cas9 variants with both the dual advantages of low off-target effect and high cleavage rate have been developed, which can reduce the off target efficiency by a factor of thousands, making the CRISPR/Cas9 system safer and efficient (78). In addition, related studies have also developed a novel type of ionizable lipid nanoparticle and extracellular vesicles as delivery vehicles (79, 80). These studies will enhance the prospect of using CRISPR/Cas9 mediated genome editing for disease treatment.

## Discussion

From the current study, we review and summarize the findings of some lncRNAs that have been implicated in SZ. These lncRNAs may not only play important roles in the pathogenesis of SZ, but also serve as potential biomarkers and therapeutic targets for this disease in the future.

Currently, some dysregulated lncRNAs in SZ patients have been identified to play key roles in its pathogenesis. However, the biological functions and mechanisms of lncRNAs in SZ are still preliminary and need to be further explored. This will facilitate further understanding of the pathogenesis of SZ as well as the development of new diagnostic and therapeutic strategies. In addition, genetic mutations in lncRNAs themselves are also involved in the pathogenesis of SZ and increase the risk of SZ, but the specific mechanism needs to be further explored. It is worth noting that aberrant methylation and mutation of lncRNAs genes may be responsible for dysregulated expression of lncRNAs in SZ patients (44, 81, 82).

The current study demonstrated that lncRNAs in peripheral circulating blood could serve as biomarkers for clinical diagnosis and prognosis of SZ. In addition, In addition, recent studies have also found that lncRNAs capture features of SZ tissue more accurately than mRNAs, and machine learning of RNA-seq data from dorsolateral prefrontal cortex (DLPFC) found an average prediction accuracy of 67% for SZ patients based on coding genes and 96% for SZ patients based on lncRNAs (83). But there are still some problems and challenges regarding the study of lncRNAs as biomarkers for SZ: (1) The study designs regarding lncRNAs as biomarkers for SZ are discovery studies with small clinical samples and lack validation studies; (2) The expression of lncRNAs has developmental stage and tissue or cell type specificity. That is, the expression of lncRNAs is spatiotemporal (84–86). It also has characteristics that are influenced by other factors such as gender, medications as well as comorbidities. It is therefore also critical to select suitable and standardized clinical samples; (3) It is noteworthy that recent studies have found differences in the expression levels of lncRNAs in serum exosomes of patients with SZ compared with healthy controls (87). Considering that exosomes can cross the BBB and transfer from the central to the peripheral blood (88). The identification of brain-derived lncRNAs may be the key to explore potential lncRNAs biomarkers in SZ; (4) Since there are many similarities of clinical symptoms between SZ and other psychiatric disorders, it is of great clinical importance to possess SZ specific biomarkers, especially to improve the efficiency of disease diagnosis. Although three lncRNAs were identified in PBMCs of SZ patients and might be potential biomarkers for distinguishing SZ from MDD and GAD (48), whether it could also distinguish SZ from other psychiatric disorders (such as bipolar disorder or autism) remains to be further explored; (5) Due to the diversity of pathogenic factors and the heterogeneity of clinical manifestations in SZ (89), it is possible that single lncRNAs may not work well as biomarkers. Therefore, combining lncRNAs with other biomarkers may be considered to improve the ability of diagnosis and prognosis; (6) The discovery of lncRNAs in the amygdala region of SZ patients that can be used to distinguish subtypes of SZ suggests that lncRNAs may serve as biomarkers for SZ subtypes (21), but currently no subtype grouping has been performed when exploring lncRNAs expression in the peripheral blood of SZ patients, which deserves further exploration in the future.

So far, although most studies on therapeutic lncRNAs targeting are still in the preclinical stage (90), and no studies have reported the therapeutic effects of targeting lncRNAs in SZ, relevant studies have indicated that lncRNAs can serve as potential therapeutic targets for SZ. In recent years, there have been a variety of therapeutic strategies for lncRNAs as therapeutic targets, including small interfering siRNAs, ASOs and CRISPR/Cas9. But these treatment strategies have some safety and technical problems in itself, moreover, the etiology of SZ is considered to be multifactorial, so the effectiveness regarding lncRNAs as therapeutic targets for SZ cannot be overestimated, and future studies may consider combining lncRNAs with other therapeutic agents to enhance the therapeutic efficacy.

In conclusion, lncRNAs are involved in the pathogenesis of SZ and provide a new approach for disease diagnosis and treatment, which is of great clinical research value. However, the current study is still in the preliminary stage, and it deserves further investigation in the future.

## Author contributions

GW was responsible for reviewing the literature and writing the manuscript. XD, ZL, YD, JL, and XL participated in providing ideas for the article. SL and YX participated in revising the manuscript. All authors contributed to the article and approved the submitted version.
